# A computational suite for the structural and functional characterization of amyloid aggregates

**DOI:** 10.1016/j.crmeth.2023.100499

**Published:** 2023-06-12

**Authors:** Zengjie Xia, Yunzhao Wu, Jeff Yui Long Lam, Ziwei Zhang, Melanie Burke, Emre Fertan, Rohan T. Ranasinghe, Eric Hidari, John S.H. Danial, David Klenerman

**Affiliations:** 1 Yusuf Hamied Department of Chemistry, University of Cambridge, Cambridge CB2 1EW, UK; 2 UK Dementia Research Institute, University of Cambridge, Cambridge CB2 0AH, UK

**Keywords:** Image processing, protein aggregate characterisation, neurodegenerative disease, super-resolution imaging, fluorescence microscopy, analysis automation

## Abstract

We developed the aggregate characterization toolkit (ACT), a fully automated computational suite based on existing and widely used core algorithms to measure the number, size, and permeabilizing activity of recombinant and human-derived aggregates imaged with diffraction-limited and super-resolution microscopy methods at high throughput. We have validated ACT on simulated ground-truth images of aggregates mimicking those from diffraction-limited and super-resolution microscopies and showcased its use in characterizing protein aggregates from Alzheimer’s disease. ACT is developed for high-throughput batch processing of images collected from multiple samples and is available as an open-source code. Given its accuracy, speed, and accessibility, ACT is expected to be a fundamental tool in studying human and non-human amyloid intermediates, developing early disease stage diagnostics, and screening for antibodies that bind toxic and heterogeneous human amyloid aggregates.

## Highlights

• ACT is an automated computational suite for fluorescence microscopy image analysis
• It achieves 90% counting accuracy for aggregates with fast processing times
• ACT is user friendly, Python based, and applicable to human brain disorder research

## Motivation

Protein aggregation is a common feature of many neurological disorders, such as Alzheimer’s and Parkinson’s diseases. Aggregates produced at early disease stages are cytotoxic, nanoscopic, structurally heterogeneous, and present in human samples at low concentrations.^14^ Therefore, detection and characterization of protein aggregates at high throughput is a fundamental prerequisite to understanding, diagnosing, and curing neurodegenerative diseases. Single-molecule microscopy and microscopy-based assays can visualize single-protein aggregates with nanometer resolution and quantify their ability to permeabilize lipid membranes with high sensitivity. A computational tool to characterize those aggregates does not exist.

## Graphical abstract

**Figure:**
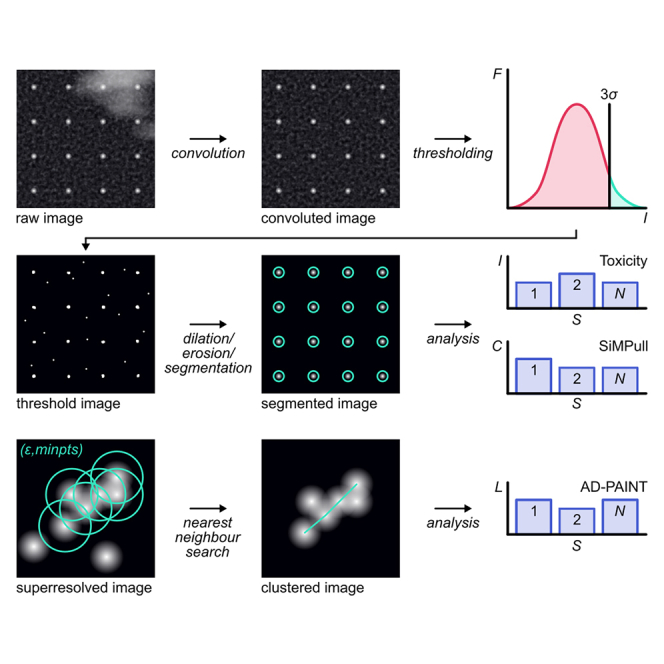

## Teaser

Xia et al. develop ACT, a user-friendly Python software, for accurate counting and characterization of aggregates using fluorescence microscopy. Validated on recombinant and human-derived aggregates, ACT’s speed and accessibility make it an essential tool for protein aggregate image analysis.

## Introduction

Neurodegenerative disorders, such as Alzheimer’s and Parkinson’s diseases (AD and PD, respectively), are characterized by the aberrant misfolding and aggregation of intrinsically disordered proteins.^1^^,^^2^^,^^3^ Misfolded or intrinsically disordered proteins aggregate into soluble oligomers that later form insoluble filaments, which deposit in the brain. Traditionally, insoluble filaments were observed as the main causative agent behind cognitive decline. However, and more recently, soluble oligomers, formed earlier in disease, were found to correlate with loss in brain function.^4^ Although the structure of insoluble aggregates was determined using cryoelectron microscopy (cryo-EM),^5^^,^^6^ characterization of the toxic, disease-causing, and soluble aggregates remains challenging owing to their scarcity in human samples, their nanoscopic size, and their heterogeneous structure. We have previously developed three methods to characterize soluble aggregates in human samples.^7^^,^^8^^,^^9^

**Figure 1:**
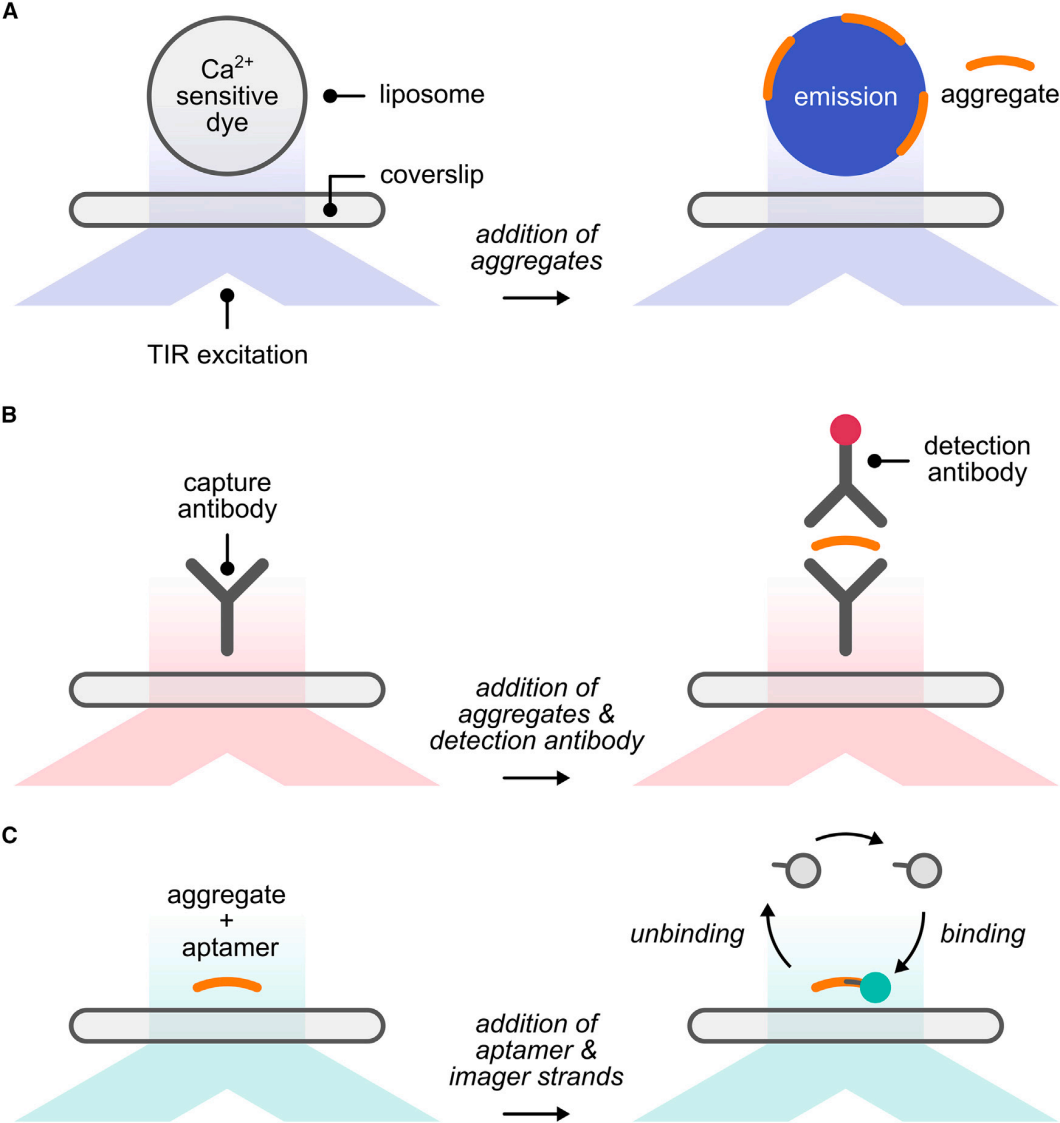
Schematic of the three microscopy methods used to measure the membrane permeability, number, and length of amyloid aggregates (A) An assay to quantify the toxicity of aggregates by measuring their ability to permeabilize surface-tethered liposomes. The liposomes are loaded with a calcium-sensitive dye, which fluoresces when the liposomes are permeabilized and calcium ions in the surrounding solutions diffuse inside. The intensity of fluorescence emission correlates with the degree of permeabilization. The liposomes are excited under total internal reflection (TIR) of the excitation laser beam to ensure that only the emission from the liposomes is detected within a shallow (<300 nm), surface-penetrating evanescent field to produce high signal-to-noise ratio images. (B) A single-molecule pull-down (SiMPull) assay to measure the number of aggregates in a solution. Surface-tethered “capture” antibodies bind free-floating aggregates in a solution, which are then detected using “detection” antibodies. Unlike the capture antibodies, the detection antibodies are DNA or fluorescently labeled, and direct fluorescence or DNA-PAINT can be observed when the detection antibodies are excited by the evanescent field of the TIR excitation, allowing single-aggregate detection with a high signal-to-noise ratio. The detection antibody can be different from the capture antibody to detect different aggregate isoforms. (C) An aptamer-based DNA-PAINT (AD-PAINT) assay to super-resolve aggregates and measure their lengths. Aptamers in solution bind to aggregates adsorbed on a surface. Short (six to nine bases) and fluorescently labeled (e.g. cy3B) “imager” strands in solution transiently bind to complementary “docking” strands appended from the aptamers. Therefore, a stochastic subset of the molecules-of-interest is lit up at a time under TIR excitation. With repeated cycles of imaging, the molecules of interest can be reconstructed and resolved in nanometer precision. The super-resolved image shows the aggregates with a much higher (∼10×) resolution compared with conventional fluorescence microscopy. Not to scale.

The first method is an ultra-sensitive, high-throughput microscopy-based assay to measure the ability of protein aggregates to disrupt lipid membranes (Figure 1A).^7^ In this method, liposomes loaded with a calcium-sensitive dye are tethered onto a glass coverslip, and a solution of calcium salt and protein aggregates is loaded on top. Aggregates that can permeabilize lipid membranes lead to a local calcium ion influx and result in localized fluorescence emissions. The intensity of those emissions correlates with the permeability of the liposomes, and therefore, by measuring the intensity of those emissions, the ability of aggregates to permeabilize lipid membranes can be precisely quantified and compared between different samples.

The second method is a variant of the single-molecule pull-down assay (SiMPull), a microscopy-based assay to measure the number of aggregates in a biological sample with a single-aggregate sensitivity (Figure 1B).^10^ In this variant of SiMPull, a protein aggregate is sandwiched between two antibodies: one coverslip-affixed antibody that captures the aggregate and a second fluorophore-conjugated antibody used for fluorescence detection. Aggregates immuno-adsorbed onto the glass coverslip are detected as diffraction-limited spots, which can be accurately counted to infer aggregate concentration in a sample of interest. SiMPull can detect aggregates at low concentrations (i.e., a few aggregates above a blank measurement) and is thus compatible with samples where such species are scarce.

The third method is a variant of the super-resolution microscopy method known as DNA point accumulation in nanoscale tomography (DNA-PAINT; Figure 1C).^11^ Conventional optical microscopy is limited in resolution to ca. 200 nm due to the diffraction of light. DNA-PAINT can overcome this limit, allowing sub-20 nm resolution imaging. In DNA-PAINT, single fluorophores are switched on and off, one after the other, using short and complementary DNA strands. “Docking strands” labeling a sample and fluorophore-conjugated “imager strands” in solution transiently bind and unbind. When a fluorophore is bound (i.e., on), its center can be estimated with nanometer precision. These precise locations are registered on a map to show a sample with higher clarity. We developed a variant of DNA-PAINT called aptamer DNA-PAINT (AD-PAINT) that is exclusively used to super-resolve nanoscopic aggregates.^9^ AD-PAINT relies on the adsorption of an aggregate to a coverslip. Detection of the aggregate is performed using a single-strand DNA aptamer, part of which forms a secondary structure and recognizes β-sheet structures in an aggregate and another part of which acts as a docking strand for free-floating, fluorophore-conjugated imager strands in solution. AD-PAINT was shown to super-resolve aggregates from several disease models as well as human biofluids and postmortem brain punches.^9^^,^^12^ Aggregates can also be captured on a SiMPull assay and super-resolved by conjugating the detection antibody with a docking strand to allow DNA-PAINT measurements. Furthermore, aggregates can be super-resolved using a similar modality, known as direct stochastic optical reconstruction microscopy (dSTORM), which only requires the conjugation of an antibody with a photoswitchable fluorophore.^13^ As such, structurally heterogeneous and polymorphic human aggregates can be super-resolved with nanometer resolution.

Using the above methods, we have shown human and synthetic aggregates of different sizes to exhibit different neurotoxic and inflammatory properties.^12^^,^^14^^,^^15^ However, and despite being well established, the above methods are not widely accessible, as all data acquired using these methods are processed and analyzed in house. To overcome this limitation and allow wider adoption of these single-aggregate methods, we have developed the aggregate characterization toolkit (ACT). The ACT is a Python-based computational suite that performs three main functions: quantifies the ability of aggregates to disrupt lipid membranes from microscopy-based liposome assays, measures the number and therefore the concentration of aggregates in biological samples from SiMPull assays, and measures the length, eccentricity, and area of single, nanoscopic aggregates imaged using super-resolution microscopy. The ACT is developed for batch analysis of large datasets acquired under different conditions (i.e., from different sources—patients or models—using different antibodies, etc.) to accelerate biomedical inference based on super-resolution imaging of nanoscopic protein aggregates.

## Results and discussion

In the module that quantifies the ability of aggregates to permeabilize lipid membranes, images for a liposome assay are processed using a custom-written Python script. Three image stacks taken before the addition of sample (F_Blank_), after incubation with sample (F_Sample_), and fully activated with ionomycin (F_Ionomycin_) are averaged and aligned by cross-correlation. Liposomes are located in the ionomycin image by applying a maximum filter, and only peaks with intensity above background plus a pre-set threshold are retained. The integrated intensities for all pixels within a radius of 3 pixels from the located peaks are measured in the three images. Finally, the percentage calcium influx for each liposome is calculated using the following formula:

$$\%\text{Influx}=\frac{F_{\text{Sample}}-F_{\text{Blank}}}{F_{\text{Ionomycin}}-F_{\text{Blank}}}\times 100\%\text{.}$$

**Figure 2:**
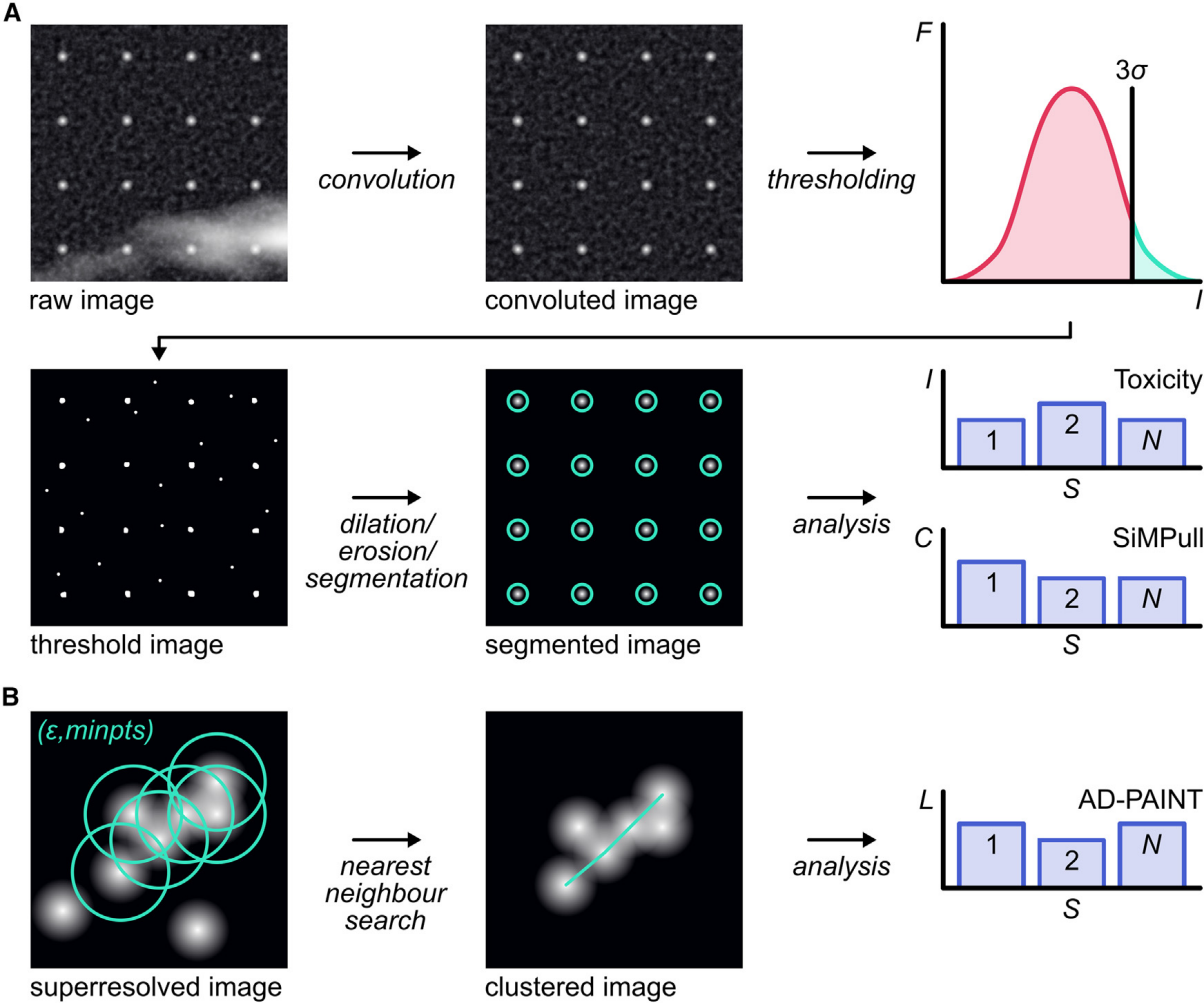
Schematic of the analysis pipeline of the ACT software (A) A diffraction-limited image is convolved with a top-hat Gaussian kernel, which is then intensity thresholded to produce a binary mask that is then dilated and eroded to produce a mask with the aggregates and aggregate-bound liposomes manifesting as bright connected regions. These regions are segmented and their integrated intensities (Is) and count (C) are calculated to infer the calcium influx and concentration of aggregates in a sample of interest. (B) A super-resolved image undergoes a density-based scan (DBSCAN) to identify aggregates (technically called clusters) from closely located localizations based on two parameters (ε, or epsilon, which dictates the search radius, and minpts, or minimum number of localizations, which dictates the minimum number of localizations within a radius epsilon to count as belonging to same cluster). After discarding the non-clustered localizations, the image of each cluster was firstly skelentonized into a “chain” of single pixels. The length of the cluster was detemined as the total length of all branches, which calculated by running nearest neighbour algorithm.

The aggregate counting module (Figure 2A) provides two methods: ComDet^16^ (a Fiji^17^-based plugin) and PyStar (a custom-written Python-based plugin). PyStar and ComDet are open-source algorithms that are used to segment bright spots on a dark background. An image is first filtered using a top-hat function to suppress the background. The filtered image is then convolved with a Gaussian kernel (in ComDet) or a Ricker wavelet kernel (in PyStar) to segment spatially separated aggregates from noise and clustered aggregates. The intensity of each pixel in the convolved image is pooled into a histogram whose mean and standard deviation are calculated. These parameters define an intensity threshold that is then used to segment aggregates from noise. The segmented image is eroded (i.e., shapes in the image are reduced in size) and dilated (i.e., the reduced shapes are expanded) to remove small areas of bright pixels, thus ensuring substantial elimination of noise and imaging artifacts. In ComDet, the processed image is then converted into a binary mask in which bright connected regions are identified and counted to output the number of aggregates. For both methods, particles detected close to the edge of an image are removed (2% of the pixels from each side for ComDet and five pixels for PyStar), to retain only particles entirely within the image.

The aggregate structural characterization module (Figure 2B) allows the user to choose between two Fiji-based plugins: ThunderSTORM^18^ and GDSC-SMLM,^19^ two robust and widely used super-resolution image processing tools. These tools produce a list of localizations that are then spatially clustered using density-based spatial clustering of applications with noise (DBSCAN), a well-established data clustering algorithm.^20^ The parameters for DBSCAN (i.e., the minimum neighboring points for a core point [minPts] and the radius for density evaluation [ε]) can be set by the user based on their requirement and image characteristics. Non-clustered localizations, arising from unspecific binding, are discarded, and those belonging to clusters are retained. The number of localizations, area, convex hull area, major axis length, and eccentricity of each cluster are calculated. In addition, the length of each cluster is calculated by skeletonizing each cluster and performing a nearest-neighbor search operation. These parameters are, finally, tabulated for further plotting and analysis.

**Figure 3:**
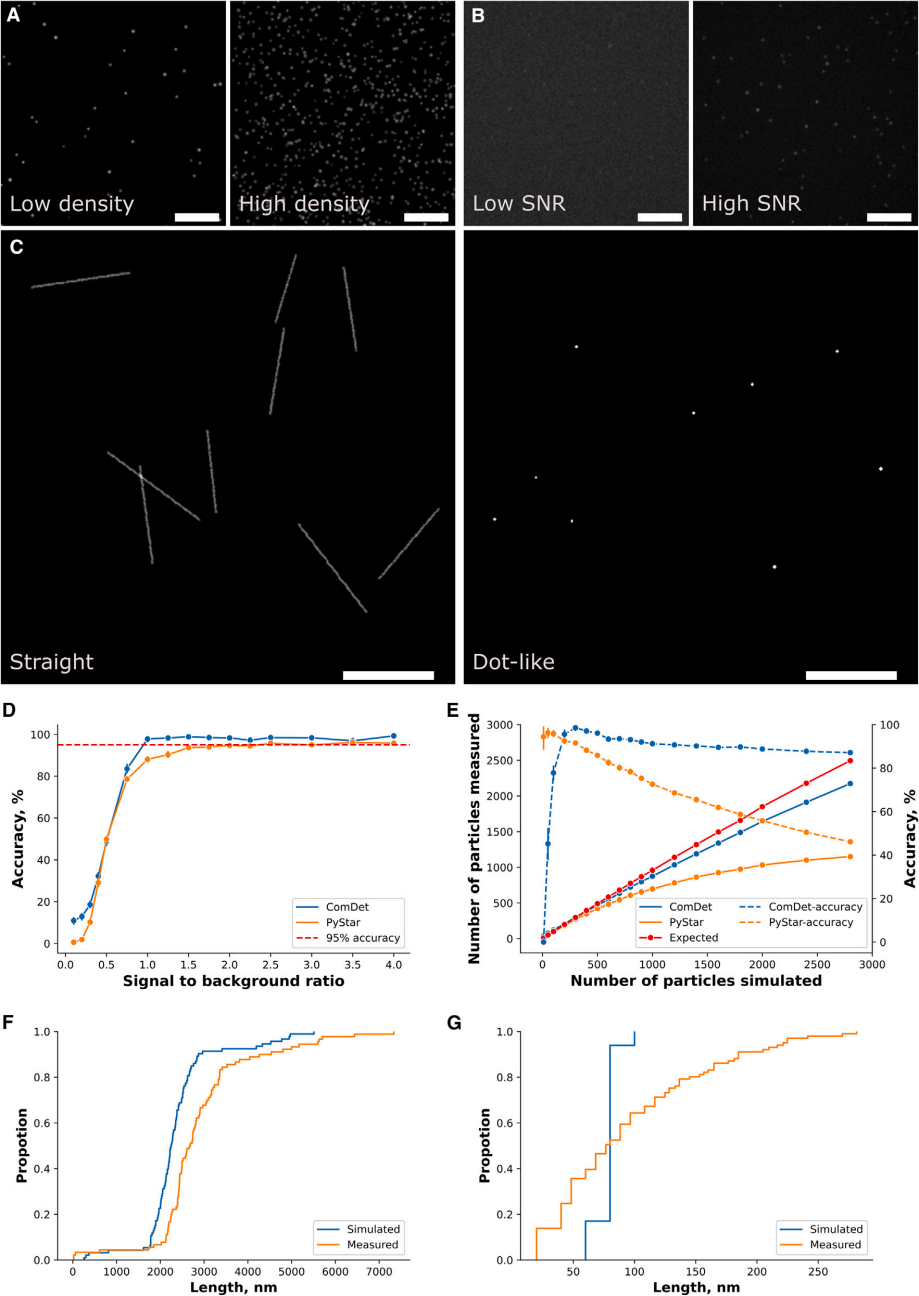
Characterizing the performance of the ACT using simulated images (A) Exemplary low-density (200 particles in a 512 × 512 pixel image) and high-density (2,800 particles in a 512 × 512 pixel image) aggregates mimicking those imaged using conventional, diffraction-limited, SiMPull microscopy. Scale bar: 50 pixels. (B) Exemplary low signal-to-noise ratio (SNR; 0.125× signal to background) and high SNR (4× signal to background) images of aggregates mimicking those imaged using conventional, diffraction-limited, SiMPull microscopy. Scale bar: 50 pixels. (C) Ground-truth structures used to simulate the super-resolved images of aggregates as imaged using super-resolution, AD-PAINT microscopy. Scale bar: 2 μm; simulated pixel size: 20 nm/pixel. (D and E) Accuracy in counting aggregates at different (D) SNRs and (E) densities imaged using diffraction-limited microscopy using the ComDet and PyStar modules. The number of particles refers to the averaged detection per FOV from 9 different simulated FOVs. (F and G) Cumulative proportion plots of simulated and measured (F) straight and (G) dot-like aggregates’ lengths.

We firstly validated ACT on simulated ground-truth images of the aggregates observed with SiMPull and AD-PAINT. For diffraction-limited images of aggregates imaged using SiMPull, we simulated aggregates at different (1) densities (from 10 to 2,800 dot-like particles in 512 × 512 pixel images; Figure 3A) and (2) signal-to-background ratios (from 0.1× to 4× above the background; Figure 3B). For super-resolved images of aggregates imaged with AD-PAINT, we simulated aggregates of different lengths (from less than 40 nm to 4 μm at a pixel size of 100 nm/pixel) and shapes (both straight and dot-like; Figure 3C) to represent filamentous and oligomeric aggregates. The performance of the ACT in counting the number of aggregates was evaluated with diffraction-limited images. For each simulated condition, nine sets of images (representing nine fields of view [FOVs]) were generated. Each set of images contained an ideal image without noise and a corresponding Gaussian-blurred simulated image. The actual number of dot-like particles simulated was counted in the ideal images (N_Simulated_) and compared with the number of particles detected with ACT (N_Measured_). The accuracy was calculated using the following formula:

$$\text{Accuracy}=\left(1-\frac{{|N}_{\text{Measured}}-N_{\text{Simulated}}|}{N_{\text{Simulated}}}\right)\times 100\%\text{.}$$

Likewise, the performance of the super-resolution image analysis was evaluated by comparing the measured aggregate length with the simulated ground truth.

The accuracy in counting the number of simulated aggregates was over 90% across a wide range of signal-to-noise ratios (SNRs) (Figure 3D) and densities (Figure 3E). For optimal analysis performance, a signal-to-background ratio greater than 1:1 (i.e., 2× signal to noise) was recommended, which would give an accuracy higher than 95%. Comparing ComDet and PyStar, the former showed a better ability to distinguish crowded particles, while the latter performs better with images with low particle density. The relatively low performance of PyStar at high particle densities is presumably because several adjacent particles are recognized as one particle, resulting in a lower number of particles, whereas ComDet is less affected by this issue with well-estimated particle size. Furthermore, the error in measuring the median length of simulated aggregates was 428 nm (19%) for the straight aggregates and 3.44 nm (4.3%) for the dot-like aggregates (Figure 3F). These results demonstrate the high accuracy of ACT in counting and sizing structurally heterogeneous aggregate structures.

**Figure 4:**
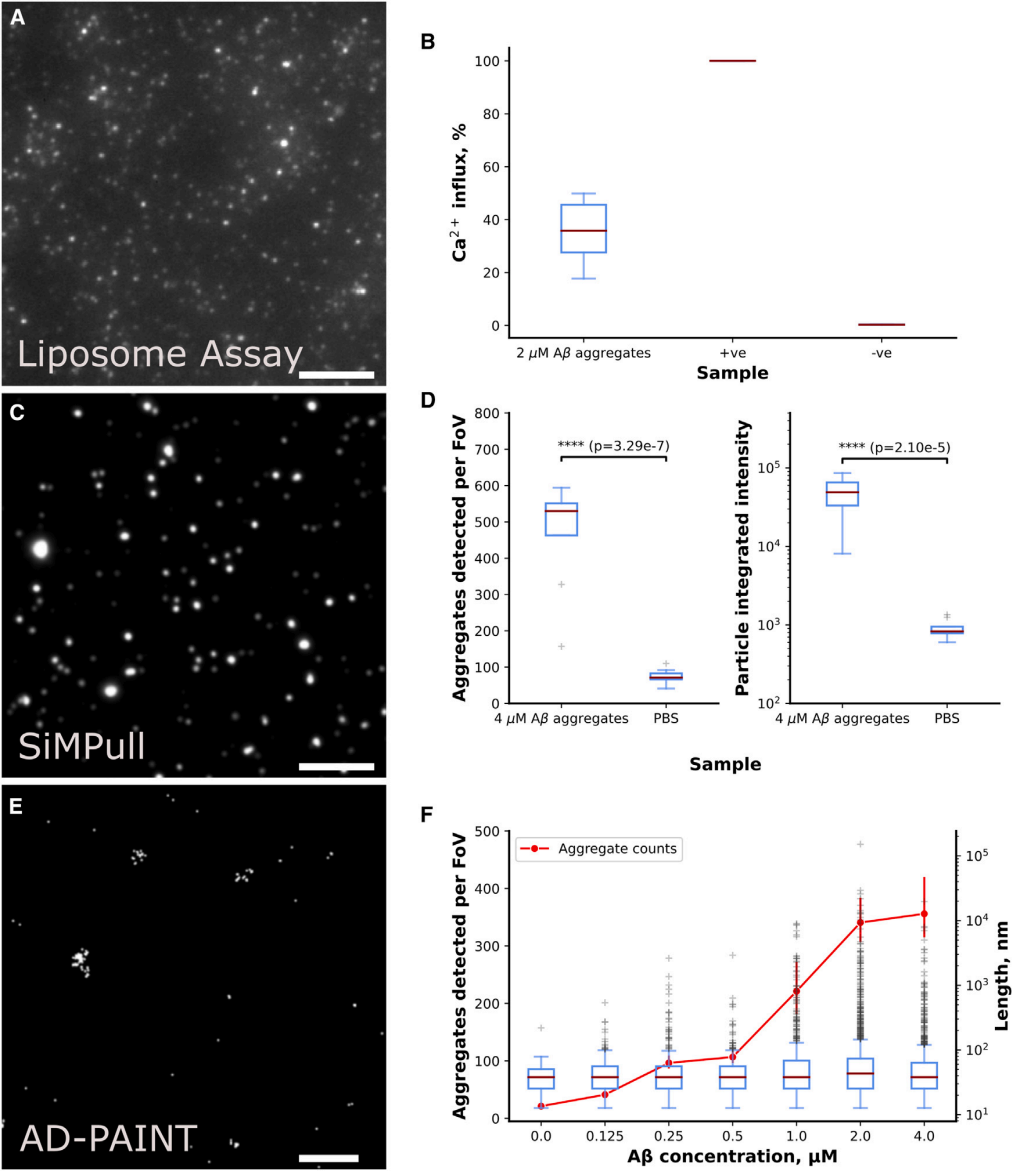
Characterizing the performance of ACT with recombinant Aβ aggregates (A) Exemplary images of liposomes exposed to Aβ aggregates. Scale bar: 5 μm. (B) Influx of calcium ions into liposomes in the presence of PBS only (negative control), ionomycin (positive control), and 2 μM Aβ aggregates. (C) Aβ aggregates pulled down to a surface and imaged using diffraction-limited microscopy. Scale bar: 5 μm. (D) Number of aggregates detected per FOV and the integrated intensity of Aβ aggregates imaged with SiMPull. (E) Aβ aggregates pulled down to a surface and imaged using super-resolution microscopy. Scale bar: 1 μm. (F) Length of aggregates super-resolved using AD-PAINT and the number of aggregates detected from each FOV at several concentrations. See also Figure S1 for statistics on the length of aggregates.

We then validated ACT on recombinant aggregates of amyloid-β (Aβ; a peptide involved in AD). We produced these aggregates at different monomeric concentrations (from 0 to 4 μM) to model the small aggregates formed at the early disease stages and the insoluble, filamentous aggregates formed at the late disease stages. We first measured the membrane permeability of 2 μM of Aβ aggregates using our single-liposome permeabilization assay (described earlier) and analyzed the results using the ACT (Figures 4A and 4B) together with a negative control (PBS only) and a positive control (1 mg/mL ionomycin: a pore-forming toxin). The median liposome influx measured for the negative control was 0.3%, positive control was 100%, and the Aβ sample was 35.8%. These results demonstrate the large dynamic range in the sensitivity of the liposome assay measurements using the ACT. We then imaged 0 and 4 μM Aβ aggregates using a SiMPull assay, and both the number of aggregates detected per FOV and their integrated intensities were analyzed using the ACT. Our measurements produced distinct populations in both the number of particles detected per FOV and the integrated intensities of these particles (shown in Figures 4C and 4D). The median integrated intensity of the 530 particles in a sample containing 4 μM Aβ aggregates was measured as 48,985. In contrast, that of the 71 particles in the negative control (i.e., a sample without aggregates) was 829. Therefore, the difference of the integrated intensity can be readily differentiated by a user-defined intensity threshold. We finally super-resolved sonicated Aβ aggregates at a wide range of concentrations (from 0 to 4 μM) using AD-PAINT and counted the number of aggregates as well as measured their length using the ACT (Figures 4E and 4F). We found that the median length of Aβ aggregates was about 40 nm for all samples. About 60% of the sonicated Aβ42 fibrils were smaller or equal to 45 nm, while 95% of the fibrils were smaller or equal to 200 nm (Figure S1). This was in agreement with the published values measured with a transmission emission microscope (50% of the fibrils were smaller than or equal to 45 nm, and 90% of the fibrils were smaller than or equal to 200 nm).^21^ This demonstrated the suitability of the ACT for the structural characterization of amyloid aggregates.

**Figure 5:**
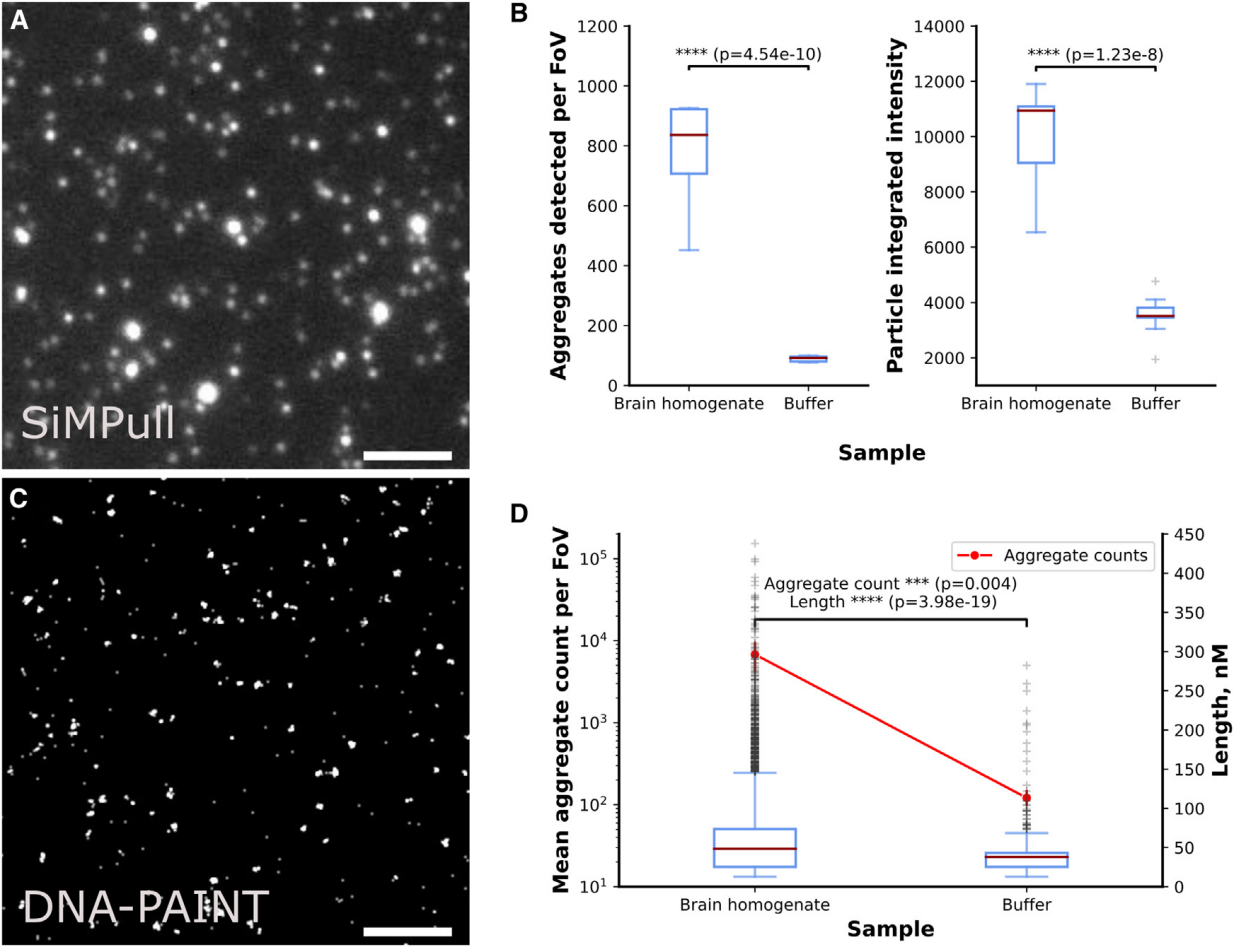
Characterizing the performance of the ACT with *ex vivo* Aβ aggregates from postmortem brain homogenates (A) Exemplary images of Aβ aggregates pulled down to a surface and imaged using diffraction-limited microscopy. Scale bar: 10 μm. (B) The number of aggregates detected per FOV and integrated intensity of human Aβ aggregates imaged with SiMPull against a negative control (brain homogenate buffer). (C) Aβ aggregates pulled down to a surface and imaged using super-resolution microscopy. Scale bar: 10 μm. (D) The number of aggregates detected per FOV and the length of aggregates super-resolved using DNA-PAINT against a negative control (brain homogenate buffer).

Having validated the ACT on simulated and recombinant aggregates, we then measured the number and length of Aβ aggregates from homogenized postmortem human brains. The number of aggregates detected using both SiMPull and DNA-PAINT showed a statistically significant difference between the brain homogenate sample and homogenization buffer. The length of Aβ aggregates in brain homogenate also has a distinct distribution from the non-specific binding detected in the buffer (Figure 5). Interestingly, the aggregates present in brain homogenate had a median length of 48 nm, with 48% of them smaller or equal to 45 nm and 99% of them smaller or equal to 200 nm, which is similar to the sonicated recombinant Aβ42 fibrils sample.

We have developed the ACT to characterize the functional and structural properties of recombinant and human-derived aggregates imaged with single-molecule microscopy methods. We validated the ACT on simulated, recombinant, and human-derived aggregates. We have shown that the ACT can achieve counting and sizing accuracy up to 90% under widely used experimental conditions. The ACT is written in Python and is developed with a user-friendly GUI centered around batch processing. Furthermore, the ACT is fast; using an 12^th^ generation Intel Core i7 (1.4 GHz) processor, the ACT can process an AD-PAINT image stack (ca. 5,000 frames, 512 × 512 pixels) in less than 5 min and a SiMPull/liposome assay image stack (ca. 50 frames, 512 × 512 pixels) in under 1 min—allowing ca. 50 human samples (3 FOVs per AD-PAINT sample and 9 FOVs per SiMPull/liposome assay sample) to be processed in a single day for AD-PAINT and a few minutes for SiMPull/liposome assay. The accuracy, accessibility, applicability, and speed of the ACT warrant its wider adoption in discovery-, diagnostic-, and therapeutic-based research of human brain disorders.

### Limitations of study

The current version of the ACT is developed with the Windows version of Fiji, and the cross-platform application is still under development.

## STAR★Methods

### Key resources table

**Table:** 

REAGENT or RESOURCE	SOURCE	IDENTIFIER
**Antibodies**		

6E10	BioLegend	Cat# 803002; RRID: AB_2564654

**Biological samples**		

AD brain tissue (Braak stage VI frontal cortex)	Cambridge Brain Bank	

**Chemicals, peptides, and recombinant proteins**		

Recombinant Aβ42 peptide	Stratech	Cat. No. A-1170-2-RPE-1.0mg

**Oligonucleotides**		

Aptamer-DS1 (GCCTGTGGTGTT GGGGCGGGTGCGTTATACATCTA)	ATDBio	
IS1-cy3B (CTAGATGTAT- cy3B)	ATDBio	
DBCO-concatDS2 (DBCO TEG- AAACCACCACCACCACCACCA CCACCACCACCACCA)	ATDBio	
ISconcat2-cy3B (TGGTGGT-cy3B)	ATDBio	

**Software and algorithms**		

μManager	Edelstein, A. D. et al.^22^	v1.4.22
Fiji	Schindelin, J. et al.^17^	Included in Git
ComDet	Katrukha E.^16^	v0.5.3
GDSC-SMLM 1	Herbert, A.^19^	Version 1.0
ThunderSTORM	Ovesný, M. et al.^18^	ThunderSTORM 1.3
SciPy	Virtanen, P. et al.^23^	SciPy 1.0
ACT	This paper	https://doi.org/10.5281/zenodo.7890730

### Resource availability

#### Lead contact

Further information and requests for resources and reagents should be directed to and will be fulfilled by the lead contact, John S. H. Danial (js2494@cam.ac.uk).

#### Materials availability

This study did not generate new unique reagents.

### Experimental model and subject details

The human brain sample used in this study (Gender: female, Age: 69 years, and Braak Stage VI) was acquired from the Cambridge Brain Bank (with the approval of the London-Bloomsbury Research Ethics Committee; 16/LO/0508).

### Method details

#### Simulation of diffraction-limited images

A number of Gaussian-shaped dots were randomly distributed on a 512x512 pixel blank image. The diameter of each dot ranged randomly from 7 to 10 pixels. The maximum intensities of all dots followed a normal distribution. For the noisy image for ACT analysis, two types of background noises were introduced to the ground-truth image. The baseline background noise, the intensities of which followed a normal distribution, was applied to all pixels in the whole image. The illumination background noise, which simulated the imaging background due to uneven illumination, followed a 2D Gaussian distribution that had higher intensities at the center of the image and lower intensities at the margins.

For the ACT density test, the total number of dots ranged between 10 and 2800. These dots were randomly distributed in the image. To eliminate the errors induced by overlapping dots, the true number of dots was counted by the ‘find maxima’ plugin in Fiji with the prominence threshold set to 1. The mean intensity of the dots was set to 3000 with a standard deviation of 600. The mean baseline noise was set to 2000 and its standard deviation was set to 200. For the SNR test, the total number of dots was set to 300 and the SNR, calculated by mean dot intensity divided by mean baseline background intensity, varied between 0.1 and 4. A Gaussian mask with a mean of 1 was applied to the simulated images to mimic the illumination from a Gaussian-shaped laser beam. In both tests, the ComDet threshold and estimated particle size were set to 3 and 2, respectively, while the PyStar threshold and the erosion size were set to 2 and 1, respectively. The accuracy of analysis with alternative parameters is presented in the supplementary information (Figure S2).

#### Simulation of super-resolved images

Round dots or line-shaped objects were randomly distributed on a 2500x2500 pixel blank image. The dot diameters and line lengths were normally distributed. All occupied pixels on the super-resolution image were considered potential blinking sites and were randomly set to ‘ON’ or ‘OFF’ to simulate the blinking of fluorophores. The positions of the ‘ON’ pixels were then recorded and used to generate a Gaussian-shaped spot on a 500x500 pixel image at the corresponding positions (i.e., the original x-y coordinates divided by 5). The spot intensity also followed a normal distribution. Background noise, the intensities of which followed a normal distribution, was added to the generated diffraction-limited image. Fiducial markers, with an intensity of 5 folds of that of the average spot intensity, were subsequently introduced to the diffraction-limited image. The above operation was repeated to generate a stack of 1000 frames of simulated, diffraction-limited images, with randomly blinking dots and fixed fiducial markers. Random drifts along the x or y direction were then introduced to all frames to simulate stage drifts during the time-lapse imaging. Finally, the image stack was analysed using either ThunderSTORM or GDSC SMLM method in ACT, followed by filtering, DBSCAN, and length calculation.

#### Preparation of sonicated recombinant Aβ42

Lyophilised monomeric recombinant Aβ42 peptide (Stratech, Cat. No. A-1170-2-RPE-1.0mg) was dissolved in PBS (pH = 7.4) at 200 μM on ice. The solution was quickly aliquoted and snap-frozen. To prepare recombinant Aβ42 fibrils, an aliquot was thawed and diluted to 4 μM in 1xPBS supplemented with 0.01% NaN_3_ (Merck, Cat. No. 71290) and incubated at 37°C under quiescent conditions for one week. The Aβ42 fibrils were then sonicated as described previously^24^ with modification. The one-week aggregated Aβ42 aliquot was immersion sonicated in an ice water bath with a 3-mm titanium probe (Sonicator microprobe 4422, Qsonica) mounted on a tip sonicator (Ultrasonic processor Q125, QSonica) at 20 kHz with 40% of power for 24×5-s bursts with 15-s rests between bursts. Thereafter, the sonicated aggregates were centrifuged, aliquoted and snap frozen. The aliquots were stored at −80°C until use.

#### Preparation of human-derived aggregates

Homogenisation buffer (50 mL) was prepared as follows: NaCl (0.8 M), EGTA (1 mM), Sarkosyl (0.1%), sucrose (10%), cOmpleteTM Protease Inhibitor (one tablet; Roche, Cat. No. 04574834001) and PhosSTOP Phosphatase Inhibitor (five tablets; Roche, Cat. No. 04574834001) were dissolved in 10 mM Tris-HCl (pH 7.4). The solution was then filtered with 0.2-μm filter and stored at 4°C.

AD brain tissue (Braak stage VI frontal cortex, Cambridge Brain Bank) was homogenised as described previously.^25^ Briefly, the AD brain tissue was homogenised at 4°C in ten volumes of homogenisation buffer. The homogenate was centrifuged at 4°C at 20,000 g for 20 min, and the upper 90% of the supernatant was retained. The pellet was re-homogenised in five volumes of homogenisation buffer, and then centrifuged at 4°C at 20,000 g for 20 min. The upper 90% of this supernatant was removed and combined with the first supernatant, and the mixture was aliquoted and frozen at −80°C until use.

#### Imaging setup for experimental validation

Four lasers operating at 405 nm (LDM-405-350-C, Lasertack GmbH), 488 nm (Toptica iBeam smart, Toptica), 561 nm (Cobolt Jive, HÜBNER GmbH & Co KG) and 638 nm (Cobolt 06-MLD-638, HÜBNER GmbH & Co KG) were coupled to the optical axis of a 1.49 N.A. 100x CFI Apo TIRF objective (MRD01991, Nikon) mounted on an inverted Ti-E Eclipse microscope (Nikon, Japan). The lasers’ powers were controlled by their corresponding software or attenuated by neutral density filters. The laser beams were then passed through the aligning mirrors and were combined by their corresponding dichroic mirror (for 405 nm: FF458- Di02-25x36, Semrock; for 488 nm: FF552-Di02-25x36, Semrock; for 561 nm: FF605-Di02-25x36, Semrock) before being focused by an aspheric lens (C220TMD-A, Thorlabs) to the square-core optical fiber (05806-1 Rev. A, CeramOptec). Launching was optimised using a free space fiber launch system (KT120/M, Thorlabs). Speckles from the fiber were removed using a vibration motor (304-111, Precision Microdrives Ltd.) mounted on a custom 3D printed mount.^26^ The combined laser beam coming out from the optical fiber was then collimated (C40FC-A, Thorlabs) and cleaned up by a quad-band excitation filter (FF01-390/482/563/640-25x36). To mechanically decouple the collimator from the microscope body, one thick (OR26 × 2V175, Hooper Ltd.) Viton O ring was inserted between the external thread of the collimator and the internal thread of the mating optomechanics to minimise mechanical vibrations. The cleaned and collimated beam was then passed through the back port of the microscope and landed on an achromatic doublet lens (AC254-125-A-ML, Thorlabs). The excitation beam was then reflected by a penta-band dichroic beam splitter (R405/488/561/635/800-T1-25×36, Semrock) and focused on the sample by the objective. The size of the excitation beam was modified by adjusting the distance between the collimator (C40FC-A, Thorlabs) and back focal plane. Fluorescence from the sample was collected by the objective and passed through a quad-band emission filter (FF01- 446/523/600/677-25x36, Semrock) and their corresponding appropriate filters (for both 405 and 488 nm induced fluorescence: BLP01-488R-25x36, Semrock and FF01-520/44-25x36, Semrock; for 561 nm induced fluorescence: LP02-568RS-25x36, Semrock and FF01-587/35-25x36, Semrock; for 638 nm induced fluorescence BLP01-635R-25x36, Semrock) mounted on a high-speed filter wheel (HF110A, Prior Scientific) before being recorded on an EMCCD camera (Evolve 512, Photometrics) operating in frame transfer mode (EM Gain of 3.1 electrons/ADU and 250 ADU/photon). Each pixel corresponded to a length of 101.2 nm on the recorded image. The microscope was also fitted with a perfect focus system (PFS) which auto-corrects the z-stage drift during a prolonged imaging period. To remove the stray infrared laser beam from the PFS, a short-pass filter (FESH0750, Thorlabs) was mounted on the entrance port of the EMCCD camera.

Slides were fixed on a microscope stage and coupled to an objective using refractive index-matched low-autofluorescence immersion oil (refractive index n = 1.518, Olympus, UK). Images were taken in a grid using an automation script (μManager v1.4.22).^22^Exposure times were set at 50 ms and 100 ms for diffraction-limited imaging and DNA-PAINT respectively. For diffraction-limited and DNA-PAINT imaging, 50 frames and 4000 frames were acquired respectively.

#### Preparation of materials for AD-PAINT

Aptamer-DS1 (GCCTGTGGTGTTGGGGCGGGTGCGTTATACATCTA), IS1-cy3B (CTAGATGTAT-cy3B), DBCO-concatDS2 (DBCO TEG- AAACCACCACCACCACCACCACCACCACCACCACCA) and ISconcat2-cy3B (TGGTGGT-cy3B) were purchased from ATDBio (Southampton, UK). They were synthesised on the 1.0 μmol scale and purified by HPLC unless otherwise stated. Dye-labelled oligonucleotides were synthesised on the 0.2 μmol scale and purified by double HPLC. Lyophilised oligonucleotides were dissolved in 18.2-MΩ·cm water (filtered by 0.02-μm filter (VWR, Cat. No. 516-1501)) to concentrations of 50–1000 μM as confirmed by A_260_, aliquoted and stored at −-20°C.

The stock solution of aptamer-DS1 (1000 μM) was diluted 10-fold into lithium cacodylate buffer (pH 7.3), which contains 1 M of KCl (Breckland, EC No. 231-211-8, Stock Code: 0001276), 0.1 M of cacodylic acid (Merck, Cat. No. C0125), and 0.1 M of lithium hydroxide (Merck, Cat. No. 909025). The aptamer-DS1 solution at 100 μM was heated to 95°C for 10 min, and then cooled down slowly overnight to room temperature.

The imaging solution for AD-PAINT was prepared as previously reported.^3^ Briefly, ultrapure grade thioflavin-T (Anaspec, Cat. No. AS-88306) was dissolved in 18.2-MΩ·cm water at 100 μM as confirmed by A_412_ (ε412 = 31600 M^−1^cm^−1^). The thioflavin-T solution was then filtered by a 0.02-μm filter (VWR, Cat. No. 516-1501), stored at 4°C in the dark for no more than one month. The final working imaging solution for AD-PAINT contains 1 nM of IS1 cy3B, 100 nM of Aptamer-DS1 and 5 μM of thioflavin-T solution in 1xPBS.

#### AD-PAINT of recombinant Aβ42 aggregates

AD-PAINT was performed as described previously.^9^ Briefly, a 50 mm-diameter round coverslip (VWR, Cat. No. 631-0178) was cleaned with argon plasma (PDC-002, Harrick Plasma) for 1 h. A 50-well PDMS gasket (Merck, Cat. No. GBL103250) was cut into halves and then affixed to the coverslip. The slide was then treated with 0.2 μm-filtered 1% Tween (Fisher Scientific, Cat. No. BP337-100, Lot No. 179118)/PBS for 1 h. It was then rinsed two times with 1xPBS (0.2 μm-filtered). Sonicated Aβ42 aggregates was then introduced onto the slide and incubated for 1 h. After incubation at room temperature, the sample was removed, and the wells were filled with the imaging solution (see previous section). To avoid evaporation over prolonged imaging, another clean coverslip was layered on top of the PDMS gasket.

#### SiMPull of Aβ aggregates in brain homogenate

The glass surface on the coverslips (VWR, Cat. No. MENZBC026076AC40) was passivated as described previously^27^^,^^28^^,^^29^ with modifications. Briefly, the coverslips were first cleaned by sonication (Ultrasonic cleaner USC100T, VWR) with a series of solvents (10 min in each of 18.2 MΩ cm water, acetone (Thermo Fisher, Cat. No. 10442631), and methanol (Thermo Fisher, Cat. No. 10675112)). The coverslips were then etched by sonication with 1 M KOH for 20 min. After that, they were rinsed with methanol, 18.2-MΩ·cm water, and methanol before dried with a stream of nitrogen. They were then cleaned with argon plasma (PDC-002, Harrick Plasma) for 15 min. The surfaces were then silanised with 3-aminopropyl triethoxysilane (Fisher Scientific UK, cat. no. 10677502), acetic acid (Merck, Cat. No. 45726) and methanol in the ratio of 3:5:100 in the sonicator for 60-s sonication with 10-min rest for two cycles. The coverslips were then rinsed with methanol, 18.2-MΩ·cm water, and methanol before dried with a stream of nitrogen. A 50-well PDMS gasket (Merck, Cat. No. GBL103250) was then affixed each coverslip. Each well was passivated by firstly introducing 9 μL of a freshly prepared 100:1 aqueous mixture of methoxy-PEG-Succinimidyl Valerate (110 mg mL^−1^, Mw ∼5,000; Laysan Bio Inc., Cat. No. MPEG-SVA-5000) and biotin- PEG-Succinimidyl Valerate (100 mg mL^−1^, Mw ∼5,000; Laysan Bio Inc., Cat. No. Biotin-PEG-SVA-5000) followed by adding 1 μL of 1 M NaHCO_3_ (pH 8.5). After overnight incubation in a humid chamber at room temperature, the coverslips were rinsed with 18.2-MΩ·cm water and dried with a stream of nitrogen. Each well was then further passivated by adding 9 μL of a freshly prepared aqueous solution of methyl-PEG4-NHS-Ester (10 mg mL^−1^; Thermo Fisher, Cat. No. 22341), followed by adding 1 μL of 1 M NaHCO_3_ (pH 8.5). The coverslips were again incubated overnight in a humid chamber at room temperature. They were then washed with 18.2-MΩ·cm water and dried with a stream of nitrogen. Finally, they were stored in a desiccator at −20°C until use.

All buffers were freshly filtered by 0.02-μm filter (VWR, Cat. No. 516-1501) before use. To each well on the biotinylated surface, 10 μL of 0.2 mg mL^−1^ NeutrAvidin (Thermo Fisher, Cat. No. 31000) in 1× PBS containing 0.05% Tween 20 (PBST) was introduced and incubated for 10 min. Each well was then rinsed twice with 10 μL of PBST and once with 10 μL of 1× PBS containing 1% Tween 20. Biotinylated 6E10 (BioLegend, Cat. No. 803007, Lot. No. B230416) were then diluted to 10 nM in 1× PBS supplemented with 0.1 mg mL^−1^ BSA (Thermo Fisher, Cat. No. B14) and 10 μL was added to each well for 10 min. Each well was then rinsed twice with 10 μL of PBST and once with 10 μL of 1× PBS containing 1% Tween 20. An aliquot of the human-derived aggregates from the brain tissue was then diluted 10-fold (v/v) with 1× PBS and 10 μL was added to each well for overnight incubation in a humid chamber at 4°C. Each well was then rinsed twice with 10 μL of PBST and once with 10 μL of 1× PBS containing 1% Tween 20. BSA was then diluted to 1 mg mL^−1^ in 1× PBS and 10 μL was added to each well for 15 min. Each well was then rinsed twice with 10 μL of PBST and once with 10 μL of 1× PBS containing 1% Tween 20. AF647-labelled (BioLegend, Cat. No. 803021, Lot. No. B304121) or DNA-labelled 6E10 (see next section) were then diluted to 500 p.m. in 1× PBS supplemented with 0.1 mg.mL-1 BSA and 10 μL was added to each well for 45 min. Each well was then rinsed twice with 10 μL of PBST and once with 10 μL of 1× PBS containing 1% Tween 20. The solution in the well was then withdrawn and another PDMS gasket was stacked on and aligned to the PDMS gasket already on the coverslip. Each well was then filled with 4 μL of 1× PBS or 1 nM of imaging strand (ISconcat2-cy3B) in PBS. Finally, a cleaned glass coverslip was layered on top of the PDMS gaskets.

#### DNA-labelling of 6E10 antibody

Antibody 6E10 (BioLegend, Cat. No. 803002) was functionalised using a SiteClick™ Antibody Azido Modification Kit (Invitrogen, Cat. No. S20026), according to the manufacturer’s instructions. Briefly, 200 μg of antibody was concentrated and buffer exchanged into the provided antibody preparation buffer. The antibody was then incubated overnight with β-galactosidase at 37°C, followed by overnight coupling to UDP-GalNAz using β-1,4-galactosyltransferase (GalT) on the next day at 30°C. The mixture was then purified by Amicon spin filter (50 kDa MWCO, Merck, Cat. No. UFC505024). The concentration of the azido-modified antibody was determined by A_280_. To the azido-modified antibody, DBCO-concatDS2 (10 M equivalents) were introduced for copper-free strain-promoted click reaction (SPAAC). The reaction mixture was then incubated overnight at 37°C. After that, excess oligonucleotide was removed by Zeba™ spin desalting columns (40kDa MWCO, ThermoFisher, Cat. No. 87766). The DNA-labelled 6E10 antibody was then further purified and concentrated by using Amicon spin filter (50 kDa MWCO). The concentration of DNA-labelled 6E10 antibody and degree of labeling were determined by A_280_ and A_260_/A_280_. The purity and degree of labeling were further confirmed using SDS-PAGE under reducing conditions (Figure S3).

### Quantification and statistical analysis

• For the validation of ACT with simulated images in Figure 3, the number of images simulated, and simulation details were written in method details. The accuracy calculation equation was stated in the results and discussion.
• Liposome assay result in Figure 4 was plotted based on 9 fields of view for each sample.
• For recombinant Aβ data in Figure 4, SiMPull results were plotted based on 9 fields of view for each sample and the statistical significance shown on the figures were calculated by T-test using SciPy^23^; number of aggregates in the AD-PAINT result was also plotted based on 9 fields of view, while the lengths were from individual aggregate (the statistics can be found in Figure S1).
• For data in Figure 5, results were plotted based on 9 fields of view for each sample and the statistical significance shown on the figures were calculated by T-test using SciPy, apart from the lengths were from individual aggregate.
• There was no exclusion of any data or subjects for the quantification and statistical analysis.

## Data Availability

• All data reported in this paper will be shared by the lead contact upon request. • Updated versions of the source code for ACT, as well as operation and installation instructions, can be obtained from https://github.com/zengjiexia/ACT (https://doi.org/10.5281/zenodo.7890730). Code for generating simulated images are also archived in the git. • Any additional information required to reanalyse the data reported in this paper is available from the lead contact upon request.

## Supplemental information

Document S1. Figures S1–S3

Document S2. Article plus supplemental information
