# Supplementary material for: A computational suite for the structural and functional characterization of amyloid aggregates
Source: Cell Rep Methods. 2023 Jun 12;3(6):100499. doi: 10.1016/j.crmeth.2023.100499 (PMC10326375; doi:10.1016/j.crmeth.2023.100499)
Supplement: Document S1. Figures S1–S3 [file mmc1.pdf]

Cell Reports Methods, Volume *3*

Supplemental information

A computational suite for the structural and functional characterization of amyloid aggregates

Zengjie Xia, Yunzhao Wu, Jeff Yui Long Lam, Ziwei Zhang, Melanie Burke, Emre Fertan, Rohan T. Ranasinghe, Eric Hidari, John S.H. Danial, and David Klenerman

**Sample**	**Length, nm**				**Proportion, %**	
	Median	***Mean***	***Min***	***Max***	***<=45 nm***	***<=200 nm***
**Sonicated Aβ42 0.125 µM**	37.95	50.21	12.65	537.97	61.82	97.58
**Sonicated Aβ42 0.25 µM**	37.95	66.57	12.65	2615.57	64.77	96.11
**Sonicated Aβ42 0.5 µM**	37.95	56.07	12.65	2915.73	64.17	97.90
**Sonicated Aβ42 1 µM**	37.95	144.44	12.65	8977.21	58.98	93.00
**Sonicated Aβ42 2 µM**	43.19	381.81	12.65	151389.87	54.00	89.95
**Sonicated Aβ42 4 µM**	37.95	110.54	12.65	19722.66	59.69	95.08
**Brain homogenate**	48.43	56.21	12.65	598.15	48.22	99.22

Supplementary Figure 1 Statistic of the size distribution of particle detected using super-resolution microscopy, related to Figure 4f.

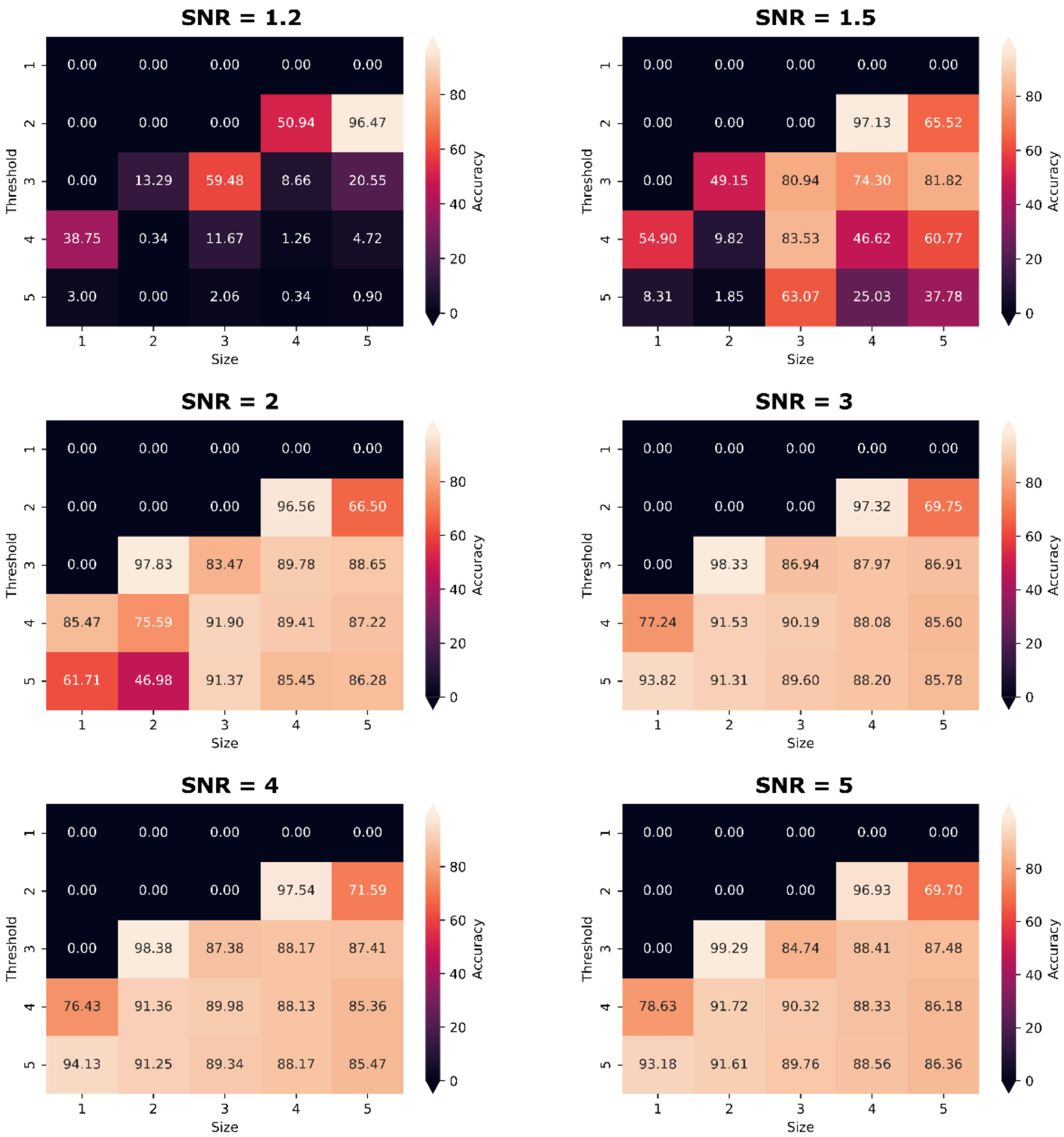

Supplementary Figure 2 Parameter evaluation for the aggregate counting module using ComDet method. (Note: Any analysis recognises number of particles that is 100% more than the ground truth would be considered as 0% accuracy, as well as the one does not detect any particle.) Related to Methods.

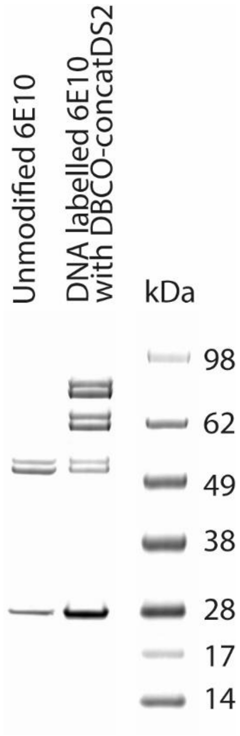

Supplementary Figure 3 Characterisation of DNA-labelled 6E10 antibody by SDS-PAGE under reducing conditions. Related to Methods.
